# Role of classic signs as diagnostic predictors for enteric fever among returned travellers: Relative bradycardia and eosinopenia

**DOI:** 10.1371/journal.pone.0179814

**Published:** 2017-06-23

**Authors:** Takashi Matono, Satoshi Kutsuna, Yasuyuki Kato, Yuichi Katanami, Kei Yamamoto, Nozomi Takeshita, Kayoko Hayakawa, Shuzo Kanagawa, Mitsuo Kaku, Norio Ohmagari

**Affiliations:** 1 Disease Control and Prevention Center, National Center for Global Health and Medicine, Tokyo, Japan; 2 Department of Infection Control and Laboratory Diagnostics, Internal Medicine, Tohoku University Graduate School of Medicine, Sendai, Japan; Institute of Tropical Medicine (NEKKEN), Nagasaki University, JAPAN

## Abstract

**Background:**

The lack of characteristic clinical findings and accurate diagnostic tools has made the diagnosis of enteric fever difficult. We evaluated the classic signs of relative bradycardia and eosinopenia as diagnostic predictors for enteric fever among travellers who had returned from the tropics or subtropics.

**Methods:**

This matched case-control study used data from 2006 to 2015 for culture-proven enteric fever patients as cases. Febrile patients (>38.3°C) with non-enteric fever, who had returned from the tropics or subtropics, were matched to the cases in a 1:3 ratio by age (±3 years), sex, and year of diagnosis as controls. Cunha’s criteria were used for relative bradycardia. Absolute eosinopenia was defined as an eosinophilic count of 0/μL.

**Results:**

Data from 160 patients (40 cases and 120 controls) were analysed. Cases predominantly returned from South Asia (70% versus 18%, p <0.001). Relative bradycardia (88% versus 51%, p <0.001) and absolute eosinopenia (63% versus 38%, p = 0.008) were more frequent in cases than controls. In multivariate logistic regression analysis, return from South Asia (aOR: 21.6; 95% CI: 7.17–64.9) and relative bradycardia (aOR: 11.7; 95% CI: 3.21–42.5) were independent predictors for a diagnosis of enteric fever. The positive likelihood ratio was 4.00 (95% CI: 2.58–6.20) for return from South Asia, 1.72 (95% CI: 1.39–2.13) for relative bradycardia, and 1.63 (95%CI: 1.17–2.27) for absolute eosinopenia. The negative predictive values of the three variables were notably high (83–92%);. however, positive predictive values were 35–57%.

**Conclusions:**

The classic signs of relative bradycardia and eosinopenia were not specific for enteric fever; however both met the criteria for being diagnostic predictors for enteric fever. Among febrile returned travellers, relative bradycardia and eosinopenia should be re-evaluated for predicting a diagnosis of enteric fever in non-endemic areas prior to obtaining blood cultures.

## Introduction

Enteric fever (typhoid and paratyphoid fever) is a systemic infection caused by human-specific food- and water-borne pathogens, such as *Salmonella enterica* subspecies *enterica* serovar Typhi or Paratyphi A, B, or C. An estimated 11.9 to 20.6 million new infections and >200,000 deaths occur annually worldwide, with the highest incidence rates in South Asia, particularly among children [1, 2]. Prevention measures including vaccination, early diagnosis, and appropriate treatment are required to manage enteric fever. However, the low isolation rate of the bacteria from blood cultures, at 40–70% [3–5], is a concerning issue, particularly in patients with prior antibiotic use. Furthermore, the lack of characteristic clinical findings and user-friendly and accurate diagnostic tools have made diagnosis of enteric fever difficult [6]. Rose spots are comparatively characteristic of enteric fever, whereas they are rarely seen in returned travellers; rose spots reportedly occur in only 4% of patients [7], because they generally develop 1 to 2 weeks after disease onset. The diagnostic usefulness of the Widal test and rapid diagnostic tests are limited due to their inaccuracy [8, 9], and polymerase chain reaction (PCR) methodology is not commercially available in Japan.

In the era that relied on physical examination, William Osler focused on patterns of pulse and fever to differentiate typhoid fever from malaria and typhus [10, 11]; however, recently, the classic signs of relative bradycardia and eosinopenia have not been considered specific diagnostic markers for enteric fever because they can also be present in other infections [12, 13]. Now that we are currently 100 years past the William Osler era, the purpose of this study was to evaluate the diagnostic usefulness of relative bradycardia and eosinopenia for predicting enteric fever among travellers returning to non-endemic areas (i.e., returned travellers).

## Methods

### Study design and setting

This matched case-control study was conducted at the National Center for Global Health and Medicine (NCGM), a tertiary care general hospital in Tokyo, Japan with 781 inpatient beds. The travel clinic in the NCGM is a GeoSentinel Surveillance Network site, which also acts as a referral hospital for returned travellers. Annually, 500 to 800 patients with travel-related illnesses visit our clinic. The data source for this study consisted of the patient database in the travel clinic and a chart review between January 2006 and October 2015.

### Ethical approval and consent

This study was approved by the Institutional Review Board at the NCGM (approval number: NCGM-G-001934-00), and was conducted according to the principles expressed in the Declaration of Helsinki. The need for informed consent was waived as this study only used the data obtained from clinical practice.

### Study population

#### Cases

**Fig 1:**
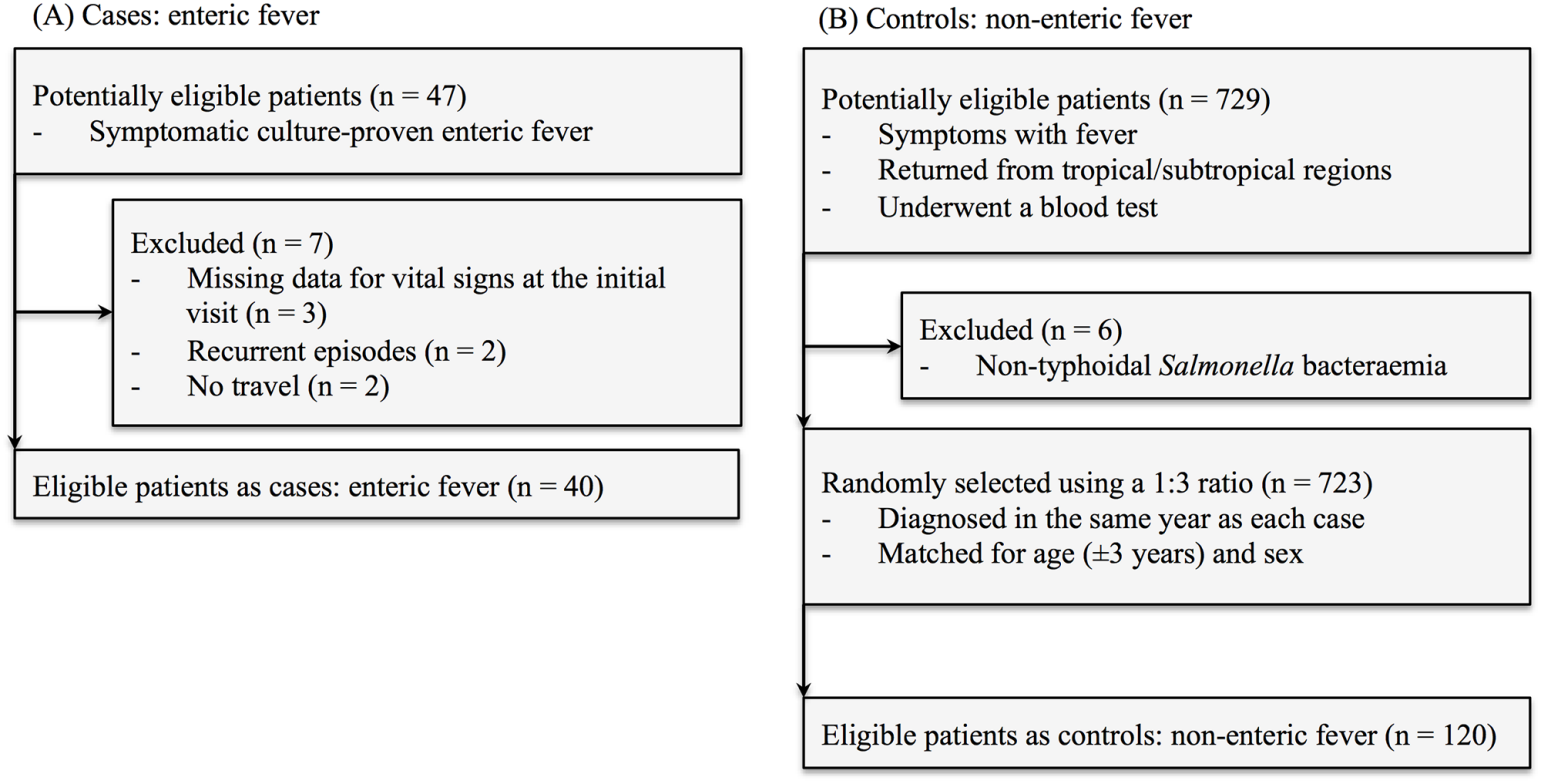
Flow diagram of selection criteria for cases (enteric fever) and controls (non-enteric fever).

All cases of symptomatic culture-proven enteric fever managed at the NCGM during the study period were included. Enteric fever was defined as isolation of *Salmonella enterica* subspecies *enterica* serovar Typhi or *S*. Paratyphi A, B, or C from the blood and/or stool of patients with fever (body temperature >38.3°C [101°F]). Among the 47 patients with culture-confirmed enteric fever, we excluded seven patients due to missing data, having more than one episode (e.g., recurrence), or no travel history (domestic cases). Finally, 40 returned travellers with enteric fever were analysed as cases (Fig 1A).

#### Controls

Patients who met all of the following inclusion criteria were considered to be potentially eligible controls (n = 729): 1) febrile (body temperature >38.3°C [101°F]), 2) having returned from travelling to tropical/subtropical regions globally, and 3) having undergone a blood test (Fig 1B). Among the 729 patients, we excluded patients with non-typhoidal *Salmonella* bacteraemia as extraintestinal focal infections, which may result in symptoms and signs similar to those of enteric fever. Furthermore, age and sex are possible confounders for enteric fever diagnosis; i.e., in endemic areas, the incidence of enteric fever is higher in children [14], and the incidence of imported enteric fever is higher in young men [7, 15]. Thus, we matched the groups for age and sex to reduce bias. The controls, matched to the cases for age (±3 years), sex, and year of diagnosis were randomly selected from the remaining 723 patients using the RAND function of Microsoft Excel, Office for Mac 2011 (Microsoft, Seattle, WA, USA) in a 1:3 ratio. As a result, 120 febrile non-enteric fever patients were analysed as controls.

### Measurements and definitions

Baseline characteristics were collected at the time of diagnosis of each infection, including age, sex, race, travel destination, and laboratory findings (eosinophil counts, etc.). Data pertaining to vital signs measured before treatment initiation, including axillary body temperature and heart rate, were also extracted from medical charts prepared on the day of diagnosis. We extracted data for any medications and underlying diseases or conditions that could affect heart rate and eosinophil count as confounders. For heart rate, we considered β-blockers, calcium channel blockers, antiarrhythmic agents, antipsychotic agents, catecholamine agonists, anticholinergic drugs, arrhythmia, ischaemic heart disease, cardiomyopathy, heart failure, sarcoidosis, amyloidosis, hemochromatosis, collagen vascular disease, neuromuscular disorder, hyper/hypothyroidism, pheochromocytoma, chronic pulmonary disease, electrolyte imbalance, anaemia, hypoxia, shock, alcohol withdrawal, and illicit drug use as confounders [16, 17]. For eosinophil count, we considered glucocorticoids, interferon alpha, myelosuppressive drugs, antihistamines, cyclosporine, leukotriene inhibitors/antagonists, phosphodiesterase inhibitors [18], atopic dermatitis, hematologic/neoplastic disorders, parasitic diseases, hypoadrenalism, collagen vascular diseases, and drug allergies as confounders.

South Asia was defined as including the following countries: Bangladesh, Bhutan, India, Nepal, Maldives, Pakistan, and Sri Lanka. Cunha’s criteria were used for relative bradycardia [12]. Namely, a patient was regarded as having relative bradycardia if the heart rate was less than each of the following criterion: 38.3°C [101°F] with 110 beats per minute (bpm), 38.9°C [102°F] with 120 bpm, 39.4°C [103°F] with 120 bpm, 40.0°C [104°F] with 130 bpm, 40.6°C [105°F] with 140 bpm, and 41.1°C [106°F] with 150 bpm. The optimal cut off value for eosinopenia is still uncertain; however, absolute eosinopenia was defined as an eosinophilic count of 0/μL, and eosinopenia was defined as ≤1% of the white cell differential, based on the previous studies [19–21]. In controls, the category of viral syndrome was defined as systematic viral infections. The following viral diseases were excluded from the category of the viral syndrome: dengue fever, haemorrhagic fever, tropical encephalitis (e.g. Japanese encephalitis), Rift Valley fever, yellow fever, and acute respiratory infections including influenza virus infection; these viral diseases were included in the present study as their own separate categories. In controls, diseases categorised in diarrhoeal disease, acute respiratory infection, and viral syndrome were generally diagnosed clinically and with cultures, not using PCR; however, all patients were monitored by follow-up visits or telephone calls until they reached an afebrile state and demonstrated clinical improvement in order to prevent misdiagnosis and overlooking of co-infections.

### Laboratory tests

Eosinophil counts were measured using the Sysmex XE-5000 (Sysmex Corporation, Kobe, Japan) automated haematology system and/or microscopic examination of stained blood films. Blood cultures were performed using the BACTEC (Becton Dickinson and Company, Franklin Lakes, NJ, USA) automatized blood culture system. Bacterial isolates from cultures were identified using the API 20E system (bioMérieux, Marcy-l'Étoile, France). Malaria was confirmed at the NCGM by a rapid diagnostic test (BinaxNOW Malaria; Alere, Waltham, MA, USA) and microscopic examination of Giemsa-stained blood smears. If the malaria parasite morphology was undetermined, nested PCR was performed, mainly for detecting non- *falciparum* malaria [22]. Dengue fever was confirmed at the National Institute of Infectious Diseases (NIID) using real-time PCR (TaqMan RT-PCR) after being screened using non-structured 1 antigen (SD Dengue Duo; Standard Diagnostics Inc., Yongin-s, Korea) and IgM-capture ELISA (Focus Diagnostics Inc., Cypress, CA, USA). Leptospirosis was confirmed at the NIID using a microscopic agglutination test, which was used for 15 serovar strains [23], and flaB-nested PCR.

### Statistical analyses

Characteristics were compared among cases and controls using chi-square (χ^2^) or Fisher exact tests for nominal variables and Mann-Whitney U tests for continuous variables. Conditional logistic regression analysis was performed to evaluate the correlations of variables with enteric fever, based on odds ratios (ORs) and 95% confidence intervals (CIs). Multivariate logistic regression analysis was used to find the independent predictors for enteric fever diagnosis. The model was not adjusted for any variables because groups were matched for age and sex, and there were no other aforementioned potential confounders to consider in the models. Diagnostic accuracy, including the sensitivity, specificity, positive/negative predictive value (PPV/NPV), or positive/negative likelihood ratio (LR) of each variable were calculated using 2 × 2 tables, and the 95% CIs were calculated by using MedCalc for Windows, Version 16.2 (MedCalc Software, Ostend, Belgium). For the binary variables, each event was presumed to be a positive test result compared to the diagnosis of enteric fever (as a gold standard). A two-tailed p-value <0.05 was regarded as statistically significant. All analyses were performed using SPSS for Windows, Version 21 (IBM Corp., Armonk, NY, USA).

## Results

### Baseline and clinical characteristics

#### Cases

*S*. Typhi was detected in 42.5% of patients (17/40), and *S*. Paratyphi A was detected in 57.5% of patients (23/40); the baseline characteristics were not significantly different between patients with typhoid fever (n = 17) and those with paratyphoid fever (n = 23) (S1 Table). Among the 40 cases, 39 (98%) were diagnosed based on blood culture results and one case with prior antibiotic use was diagnosed based on stool culture result. The median age was 31 (range 4–56) years, including a 4-year-old child and a 12-year-old child; 68% were male; and 93% were Asian (Table 1). Intergroup differences in age, sex, and ethnic origin were not significant. Of 40 cases, five (13%) had co-infections [four cases of diarrhoeal disease caused by *Giardia intestinalis*, (detected by stool microscopy), *Campylobacter jejuni*, *Shigella sonnei*, or enteropathogenic *Escherichia coli* (each confirmed by stool culture), and one case with an *influenza virus* B infection]. In total, 7.5% (3/40) of patients relapsed after initial treatment. These three relapsed patients were administered ceftriaxone monotherapy; among the patients treated with ceftriaxone alone (n = 24), the relapse rate was 12.5% (3/24). There were no fatal or severe outcomes such as death, intestinal perforation, or encephalopathy, and no chronic carriers were found after treatment.

**Table 1:** Baseline characteristics of patients with enteric fever (EF, Cases) and non-enteric fever (NEF, Controls).

Characteristics	EF (n = 40)	NEF (n = 120)	p-value
Age (years), median (range)	31 (4–56)	31 (1–57)	0.928
Male gender, n (%)	27 (68)	81 (68)	0.773
Ethnic origin, n (%)			
Asian	37 (93)	109 (91)	0.518
Japanese	36 (90)	106 (88)	0.515
non-Japanese	1 (3)	3 (3)	0.74
Caucasian	3 (8)	1 (1)	0.049
African	0 (0)	10 (8)	0.051
Travel destination, n (%)			
Southeast Asia	12 (30)	54 (45)	0.095
South Asia	28 (70)	21 (18)	<0.001
Africa	0 (0)	33 (28)	<0.001
Oceania	0 (0)	7 (6)	0.128
South America	0 (0)	3 (3)	0.419
Central America	0 (0)	1 (1)	0.75
Caribbean	0 (0)	1 (1)	0.75
Examination finding			
Relative bradycardia	35 (88)	61 (51)	<0.001
Laboratory findings, median (IQR)			
Total leukocytes (×10^3^/μL)	4.9 (3.8–6.5)	6.4 (4.1–10.7)	0.01
Eosinopenia (≤1%), n (%)	38 (95)	95 (79)	0.021
Absolute eosinopenia (0/μL), n (%)	25 (63)	46 (38)	0.008
Haematocrit (%)	40.5 (37.1–41.9)	42.6 (39–45.1)	0.003
Platelets (×10^3^/μL)	185 (145–240)	191 (145–246)	0.903
Total bilirubin (mg/dL)	0.5 (0.4–0.7)	0.7 (0.6–1.2)	<0.001
AST (IU/L)	57.5 (37.5–105)	24 (19–40)	<0.001
ALT (IU/L)	52.5 (28.8–114)	22 (15–39.5)	<0.001
LDH (IU/L)	395 (288–459)	211 (176–266)	<0.001
CRP (mg/L)	38.7 (26.1–86.9)	25.4 (7.5–57.4)	0.001

EF: enteric fever; NEF: non-enteric fever; IQR: interquartile range; AST: aspartate transaminase; ALT: alanine transaminase; LDH: lactate dehydrogenase; CRP: C-reactive protein

#### Controls

Among the 120 patients with non-enteric fever, 27% were diagnosed with a diarrhoeal disease, followed by 23% with acute respiratory infection, 18% with viral syndrome, 13% with malaria, and 11% with dengue fever (Table 2). Tropical diseases were present in 27% of controls (32/120): 13% had malaria, 11% dengue fever, 2% leptospirosis, and 1% rickettsiosis. In the 16 patients with malaria, 14 patients were infected with *Plasmodium falciparum*, one with *P*. *ovale*, and one with *P*. *malariae* (S2 Table). In the 13 patients with dengue fever, two patients had dengue shock syndrome. In the patients with a viral syndrome, two patients had infectious mononucleosis related to Epstein-Barr virus, and one had measles. Among the 120 controls, one patient with malaria had human immunodeficiency virus (HIV) co-infection and was on antiretroviral therapy, with a CD4 count of 196 cells/μL and an undetectable HIV viral load at the time of malaria diagnosis. Two patients had hypertension, including one patient that was not taking any medications and one patient taking losartan/hydrochlorothiazide. No other controls had any other co-infection or underlying diseases.

**Table 2:** Causes of infections in patients with non-enteric fever (Controls) (n = 120).

Diagnosis	Patients, n (%)
Diarrhoeal disease	32 (27)
Acute respiratory infection	27 (23)
Viral syndrome	22 (18)
Malaria	16 (13)
Dengue fever	13 (11)
Non diarrhoeal gastrointestinal diagnosis	4 (3)
Leptospirosis	2 (2)
Genitourinary infection	2 (2)
Rickettsiosis	1 (1)
Dermatologic infection	1 (1)

### Main analyses

Cases predominantly returned from South Asia (70% of cases versus 18% of controls, p <0.001; Table 1). Relative bradycardia (88% versus 51%, p <0.001) and absolute eosinopenia (63% versus 38%, p <0.001), respectively, were also more frequently noted in cases than controls. Logistic regression analysis revealed a significant association between a diagnosis of enteric fever and each predictor, including a return from South Asia (OR: 11.0; 95% CI: 4.83–25.1), relative bradycardia (OR: 6.77; 95% CI: 2.48–18.5), and absolute eosinopenia (OR: 2.68; 95% CI: 1.28–5.61; Table 3). Return from South Asia (adjusted OR: 21.6; 95% CI: 7.17–64.9), relative bradycardia (adjusted OR: 11.7; 95% CI: 3.21–42.5) and haematocrit (adjusted OR: 0.85; 95% CI: 0.76–0.95) remained independent predictors for enteric fever diagnosis even in multivariate logistic regression analysis. Furthermore, NPVs of each variable (return from South Asia, relative bradycardia, absolute eosinopenia, and eosinopenia) were remarkably high, ranging from 83.1% to 92.6% (Table 4). Notably, the NPVs of relative bradycardia and eosinopenia were 92.2% and 92.6%, respectively. However, the specificities (20.8–82.5%) and PPVs (28.6–57.1%) varied and were not high. The highest positive LR was 4.00 (95% CI: 2.58–6.20) for return from South Asia, followed by 1.72 (95% CI: 1.39–2.13) for relative bradycardia, 1.63 (95% CI: 1.17–2.27) for absolute eosinopenia, and 1.2 (95% CI: 1.07–1.35) for eosinopenia. The negative LRs were 0.36 (95% CI: 0.22–0.59) for return from South Asia, 0.25 (95% CI: 0.11–0.59) for relative bradycardia, 0.61 (95% CI: 0.40–0.93) for absolute eosinopenia, and 0.24 (95% CI: 0.06–0.97) for eosinopenia. Importantly, all of these variables met the criteria for being diagnostic predictors for enteric fever based on the positive and negative LRs; namely, each positive LR resulted in >1, and each negative LR in <1.

**Table 3:** Logistic regression analysis of variables in the prediction of enteric fever diagnosis.

Variables	OR (95% CI)	Adjusted OR (95% CI)	p-value
Return from South Asia	11.00 (4.83–25.08)	21.58 (7.17–64.93)	<0.001
Relative bradycardia	6.77 (2.48–18.46)	11.69 (3.21–42.51)	<0.001
Total leukocytes (×10^3^/μL)	1.00 (1.00–1.00)		
Absolute eosinopenia (0/μL)	2.68 (1.28–5.61)	1.77 (0.66–4.74)	0.259
Haematocrit (%)	0.91 (0.85–0.99)	0.85 (0.76–0.95)	0.003
Platelets (×10^3^/μL)	1.00 (0.95–1.05)		
Total bilirubin (mg/dL)	1.00 (1.00–1.00)		
AST (IU/L)	1.01 (1.00–1.01)		
ALT (IU/L)	1.01 (1.00–1.01)		
LDH (IU/L)	1.01 (1.00–1.01)		
CRP (mg/L)	1.07 (1.01–1.14)	1.01 (0.99–1.02)	0.067

AST: aspartate transaminase; ALT: alanine transaminase; LDH: lactate dehydrogenase; CRP: C-reactive protein; OR: odds ratio; CI: confidence interval

**Table 4:** Diagnostic predictive values of enteric fever.

Characteristics	Sensitivity (%)	Specificity (%)	PPV (%)	NPV (%)	LR+	LR-
Return from South Asia	70.0	82.5	57.1	89.2	4.00	0.36
Relative bradycardia	87.5	49.2	36.5	92.2	1.72	0.25
Absolute eosinopenia (0/μL)	62.5	61.7	35.2	83.1	1.63	0.61
Eosinopenia (≤1%)	95.0	20.8	28.6	92.6	1.2	0.24

PPV: positive predictive value; NPV: negative predictive value; LR: likelihood ratio. Diagnostic accuracy of each variable were calculated using 2 × 2 tables; each event of the binary variable was presumed to be a positive test result compared to the diagnosis of enteric fever (as a gold standard)

### Subgroup analyses

The proportion of relative bradycardia and absolute eosinopenia differed according to the diseases among controls. When comparing enteric fever to each disease (S3 Table), relative bradycardia was significantly more common in cases (88%) than controls with diarrhoeal disease (47%, p <0.001), acute respiratory infection (44%, p <0.001), and viral syndrome (41%, p <0.001), but less common in cases than controls with malaria (63%, p = 0.043) and dengue fever (85%, p = 0.56); p-values <0.01 using Bonferroni correction were regarded as statistically significant. Absolute eosinopenia was significantly more frequent in cases (63%) than in controls with acute respiratory infection (30%, p = 0.008) but less common in cases than controls with other diseases. Furthermore, when comparing enteric fever according to each disease, logistic regression analysis identified a significant association between a diagnosis of enteric fever and relative bradycardia in patients with diarrhoeal disease, acute respiratory infection, viral syndrome, and malaria but not in those with dengue fever (S4 Table). Similarly the analysis showed a significant association between a diagnosis of enteric fever and absolute eosinopenia in patients with diarrhoeal disease, acute respiratory infection, and viral syndrome but not in those with malaria and dengue fever.

## Discussion

In this matched case-control study, we evaluated the diagnostic usefulness of the classic signs of relative bradycardia and eosinopenia for predicting enteric fever among returned travellers. We found that these classic signs were more frequent in cases than controls: relative bradycardia (88% versus 51%) and absolute eosinopenia (63% versus 38%). We also found that the signs were diagnostic; the positive LR was 1.72 for relative bradycardia and 1.63 for absolute eosinopenia, and the former was an independent predictor for the diagnosis of enteric fever (aOR: 11.7; 95% CI: 3.21–42.5). These signs were not specific for enteric fever but can be useful for predicting a diagnosis of enteric fever in non-endemic areas before obtaining blood cultures among febrile returned travellers from the tropics/subtropics.

### Comparison with other studies

The present study has two important findings. First, this is the first study to use a matched case-control design to compare predictors for enteric fever diagnosis among returned travellers, particularly focusing on the classic signs of relative bradycardia and absolute eosinopenia. In recent years, these classic signs have not been considered specific diagnostic markers for enteric fever, and a little evidence was available. Relative bradycardia has been reported among patients with other tropical febrile illnesses such as malaria, dengue fever, and rickettsiosis, among others [12, 24, 25]; however, these studies have not compared quantitatively the frequency of relative bradycardia between patients with enteric fever and those with each tropical disease. A Malaysian case-control study concluded that relative bradycardia is not useful in predicting enteric fever among children [26]. However, in this previous study, the age of patients with enteric fever was higher than that of those with non-enteric fever, which can relate to heart rate in children. In contrast, our study matched the controls to the cases by age, sex, and year of diagnosis in a 3:1 ratio to reduce such bias. Tropical diseases, which may have the potential to cause relative bradycardia, were present in 27% of controls in our study; however, our study design enabled us to identify that relative bradycardia was one of the predictors for enteric fever (aOR: 11.7; 95% CI: 3.21–42.5; LR+: 1.72; LR-: 0.25). Similarly, a Turkish study concluded relative bradycardia was the diagnostic predictor for enteric fever (OR: 17.26; 95% CI: 3.20–25.59) [19], which partly supports our result. However, they did not report the specific criteria for defining relative bradycardia, although several criteria exist [12, 27]. The relative bradycardia definition used in our study was not specific for enteric fever (specificity, 49.2%), whereas, importantly, the examination signs combine the low cost and easy of use in the clinic.

Second, our study was the first to evaluate a diagnostic role of absolute eosinopenia for enteric fever, systemically. The relationship between acute infection and eosinopenia has been traditionally described in recent decades; particularly, sepsis and bacteraemia occur often with eosinopenia [28, 29]. The mechanism of decreasing the eosinophil count is considered to be, in part, secondary to sequestration of circulating eosinophils, mainly resulting from a chemotactic substance, C5a [30]. In fact, absolute eosinopenia has been reportedly observed in 72–77% of patients with enteric fever [20, 21, 31], whereas, there is little information about the usefulness of the absolute eosinopenia in predicting a diagnosis of enteric fever. Only one study in the United Arab Emirates reported that absolute eosinopenia was more frequent in enteric fever than in non-enteric fever (73% versus 25.4%) [21]. However, despite analysing a binary variable, they used the Student’s t-test, and they did not show detailed p-values or diagnostic usefulness using ORs, sensitivity and specificity, and LRs. Furthermore, the previous study included both culture-confirmed and clinically diagnosed enteric fever; 43% of cases were clinically diagnosed. In contrast, our study investigated the diagnostic accuracy of absolute eosinopenia in predicting culture-confirmed enteric fever, and suggested a possible association between a diagnosis of enteric fever and absolute eosinopenia (OR: 2.68; 95% CI: 1.28–5.61; LR+: 1.63; LR-: 0.61).

The diagnostic usefulness of each predictor, namely return from South Asia, relative bradycardia, and absolute eosinopenia, was limited owing to the lack of specificities in our study when considered independently (Table 4). In particular, the classic signs of relative bradycardia and absolute eosinopenia may not be useful to differentiate enteric fever from dengue fever (S3 and S4 Tables). Therefore, even with these predictors, considering other tropical febrile illness is required in an actual clinical setting. Furthermore, even without these predictors, it is important to obtain cultures because cultures can provide substantial information for not only patients with suspected enteric fever but also for all febrile patients.

### Study limitations

There are several limitations in this study. First, the cases included predominantly adults and Asian patients, and our controls included only one patient with rickettsiosis, while enteric fever has been historically confused with rickettsiosis. These might limit the application of these results to other populations. However, in our controls, the composition of travel-related febrile diseases was fairly similar to that of a previous study in returned travellers using the GeoSentinel Surveillance Network database between 1997 and 2006 [32]. Therefore, we believe our results represent the actual clinical setting of febrile returned travellers to non-tropical or subtropical areas, and our results are acceptable in such situations. By varying the population and geographical areas, further studies might reveal the effectiveness of these classic signs in predicting enteric fever in different settings. Second, the incubation period was not considered for each infectious disease, in either cases or controls. In clinical practice, additional information on the estimated incubation period could be useful in predicting diagnosis among afebrile returned travellers from tropics or subtropics [33]. Third, haematocrit remained an independent predictor for the diagnosis of enteric fever, even in the multivariate logistic regression analysis; however, this may be because the model was not adjusted for hypovolemic status. The median haematocrit for each disease in the controls was higher than that for enteric fever (40.5%)—that is, diarrhoeal disease (43.3%), acute respiratory infection (42.7%), viral syndrome (42.9%), malaria (41.0%), and dengue fever (41.4%). Finally, the highest risk factor for enteric fever in this study was return from travel to South Asia. Among returned travellers to developed countries, 67–85% of all patients with enteric fever were infected in South Asia [15, 34], which supports our results. However, it is also necessary to consider that the usefulness of such epidemiological information is limited when an outbreak of enteric fever occurs in areas outside of South Asia.

## Conclusions

The present study provides evidence of a diagnostic tool for enteric fever that utilises the presence of classic signs. We found that each variable showed a high NPV and each positive/negative LR met the criteria as diagnostic predictors. Hence, among febrile returned travellers to non-endemic areas, the classic signs of relative bradycardia and eosinopenia should be re-evaluated for predicting a diagnosis of enteric fever prior to obtaining blood cultures.

## Supporting information

S1 Dataset Complete dataset for the analysis. (PDF)

S1 Table Baseline characteristics of patients with typhoid fever and paratyphoid fever. (PDF)

S2 Table Clinical diagnoses for study controls. (PDF)

S3 Table Frequency of relative bradycardia and absolute eosinopenia in patients with enteric fever with (cases) and controls. (PDF)

S4 Table Logistic regression analysis of variables in the prediction of enteric fever diagnosis among enteric fever (cases) and each disease in controls. (PDF)
