# Supplementary material for: Role of classic signs as diagnostic predictors for enteric fever among returned travellers: Relative bradycardia and eosinopenia
Source: PLoS One. 2017 Jun 23;12(6):e0179814. doi: 10.1371/journal.pone.0179814 (PMC5482448; doi:10.1371/journal.pone.0179814)
Supplement: S1 Dataset — (PDF) [file pone.0179814.s001.pdf]

Case/Control	Diagnosis	Travel destination	Relative bradycardia	Absolute eosinopaenia	Eosinopaenia	Total leukocytes (×10³/μL)	Haematocrit (%)	Platelets (×10³/μL)	Total bilirubin (mg/dL)	AST (IU/L)	ALT (IU/L)	LDH (IU/L)	CRP (mg/L)
Case	*S.* Typhi	South Asia	Yes	No	Yes	6.81	36.3	245	0.3	65	35	573	12.5
Case	*S.* Typhi	Southeast Asia	No	No	Yes	1.092	40.4	187	0.4	36	22	277	50
Case	*S.* Paratyphi	South Asia	Yes	No	Yes	6.93	49.1	170	0.8	36	52	227	11.8
Case	*S.* Typhi	South Asia	Yes	No	Yes	4.93	39.8	296	0.7	19	13	219	25.2
Case	*S.* Typhi	Southeast Asia	Yes	Yes	Yes	4.69	37.5	106	0.6	68	20	520	148.1
Case	*S.* Paratyphi	South Asia	Yes	Yes	Yes	5.32	37.9	184	0.5	212	228	426	35.9
Case	*S.* Typhi and *Campylobacter jejuni*	South Asia	Yes	Yes	Yes	5.66	41.4	184	0.3	526	560	800	32.5
Case	*S.* Paratyphi and *Shigella sonnei*	South Asia	Yes	No	Yes	5.94	45.4	247	0.6	71	96	382	32.4
Case	*S.* Paratyphi	South Asia	Yes	No	Yes	4.29	35.8	226	0.3	38	29	337	10.7
Case	*S.* Typhi	South Asia	Yes	Yes	Yes	5.22	41.5	190	1	75	106	432	101.6
Case	*S.* Typhi	South Asia	Yes	Yes	Yes	4.23	41.4	174	0	28	22	238	25.1
Case	*S.* Typhi	South Asia	Yes	Yes	Yes	3.39	31.3	103	1.1	204	125	1048	200.5
Case	*S.* Paratyphi	South Asia	Yes	No	Yes	4.45	43.5	244	0.9	104	110	425	70.6
Case	*S.* Typhi	South Asia	Yes	Yes	Yes	7.26	34.8	262	0.5	164	140	404	20.8
Case	*S.* Typhi	South Asia	No	No	No	3.71	34.2	158	0.1	46	29	360	26.4
Case	*S.* Paratyphi	South Asia	Yes	Yes	Yes	7.49	41.7	221	0.2	47	44	291	76
Case	*S.* Paratyphi	South Asia	Yes	Yes	Yes	3.41	40.8	131	0.5	62	46	459	59.3
Case	*S.* Paratyphi	Southeast Asia	No	Yes	Yes	5.95	42.6	265	0.1	30	28	212	22.9

Case	*S.* Typhi	South Asia	Yes	Yes	Yes	3.23	43.6	130	0.5	49	53	402	31.5
Case	*S.* Paratyphi and enteropathogenic *E. coli*	South Asia	Yes	Yes	Yes	10.06	40.8	324	0.8	24	23	253	12.6
Case	*S.* Typhi	South Asia	Yes	No	Yes	4.8	40.1	259	0.3	77	68	403	38.5
Case	*S.* Typhi	South Asia	Yes	No	Yes	5.87	43.5	175	0.5	257	153	459	17.2
Case	*S.* Paratyphi	Southeast Asia	Yes	Yes	Yes	3.5	38.6	194	0.4	24	19	260	35.9
Case	*S.* Paratyphi	South Asia	Yes	No	Yes	7.96	41.5	397	0.6	20	29	204	16.7
Case	*S.* Paratyphi	South Asia	Yes	Yes	Yes	6.38	43	154	0.7	83	73	581	211.7
Case	*S.* Paratyphi	South Asia	Yes	Yes	Yes	4.87	36.6	131	0.5	44	43	361	57.3
Case	*S.* Typhi	Southeast Asia	Yes	Yes	Yes	11.72	38.2	127	1.9	305	356	788	108.8
Case	*S.* Paratyphi	Southeast Asia	Yes	Yes	Yes	3.41	38.3	159	0.4	50	35	300	79.8
Case	*S.* Paratyphi and influenza B virus	Southeast Asia	Yes	No	Yes	7.24	39.7	219	0.4	57	81	317	30.3
Case	*S.* Paratyphi	South Asia	No	Yes	Yes	4.76	30	205	0.3	22	16	200	63.7
Case	*S.* Paratyphi	Southeast Asia	Yes	No	No	6.73	35.2	286	0.4	231	278	540	36.9
Case	*S.* Paratyphi and *Giardia intestinalis*	Southeast Asia	Yes	Yes	Yes	9.94	41.7	238	1.1	58	62	435	175
Case	*S.* Paratyphi	South Asia	Yes	Yes	Yes	3.87	44.8	183	0.9	38	53	402	38.9
Case	*S.* Typhi	South Asia	Yes	Yes	Yes	3.36	43.6	90	1.2	109	131	387	82.8
Case	*S.* Paratyphi	Southeast Asia	Yes	Yes	Yes	4.43	36.2	149	0.5	152	148	484	144.9
Case	*S.* Typhi	South Asia	Yes	No	Yes	2.95	33.8	107	0.6	40	32	350	54.3
Case	*S.* Paratyphi	South Asia	No	No	Yes	5.57	42.8	186	0.5	108	155	477	99.1
Case	*S.* Paratyphi	Southeast Asia	Yes	Yes	Yes	4.21	41.3	110	0.7	46	28	345	121.6
Case	*S.* Typhi	Southeast Asia	Yes	Yes	Yes	3.72	40.5	120	0.7	75	83	421	126

Case	*S.* Paratyphi	South Asia	Yes	Yes	Yes	6.26	37.2	200	0.6	12	12	140	37.4
Control	Acute respiratory infection	Africa	No	No	Yes	1.197	33.9	282	0.4	49	25	318	1.2
Control	Viral syndrome	Southeast Asia	No	Yes	Yes	3.49	29.5	224	0.4	74	18	212	0.3
Control	Acute respiratory infection	Southeast Asia	No	No	Yes	7.17	34.3	230	0.5	24	10	250	11.2
Control	Malaria	Africa	Yes	No	Yes	3.87	41.1	96	2.6	155	175	520	42.8
Control	Diarrhoeal disease	Africa	No	No	Yes	12.43	42.3	325	0.7	24	12	208	2.4
Control	Diarrhoeal disease	South Asia	No	Yes	Yes	17.24	46.6	247	1.1	27	22	313	119
Control	Dengue fever	Southeast Asia	Yes	No	Yes	2.8	41	163	0.5	20	12	191	3
Control	Acute respiratory infection	Southeast Asia	No	No	Yes	14.35	40	272	0.7	21	12	323	27.7
Control	Viral syndrome	Oceania	No	No	Yes	7.91	43.9	279	0.6	19	15	186	61.4
Control	Dengue fever	Southeast Asia	Yes	Yes	Yes	1.39	46.1	109	0.6	32	22	209	7.6
Control	Viral syndrome	South America	No	No	No	8.51	43.4	300	0.5	53	60	349	5.8
Control	Diarrhoeal disease	Southeast Asia	Yes	Yes	Yes	12.87	43	211	1	18	13	202	87.4
Control	Dengue fever	Southeast Asia	Yes	Yes	Yes	3.35	41.4	124	0.7	29	17	260	26.7
Control	Diarrhoeal disease	South America	No	No	No	8.97	42	279	0.6	21	37	208	3.7
Control	Acute respiratory infection	Southeast Asia	No	No	No	5.31	38.7	169	0.6	19	13	164	2.8
Control	Diarrhoeal disease	South Asia	No	No	Yes	11.4	43.4	374	0.9	18	13	212	3.2
Control	Dermatologic infection	South Asia	No	No	Yes	5.9	37.5	353	0.6	19	15	176	12.7
Control	Diarrhoeal disease	South Asia	Yes	Yes	Yes	7.13	44.4	231	1.4	14	9	206	13.2
Control	Diarrhoeal disease	South Asia	No	No	Yes	11.77	44.3	287	1	15	9	129	19.9
Control	Diarrhoeal disease	Southeast Asia	Yes	No	Yes	8.53	38.6	267	0.1	15	14	204	9.1

Control	Diarrhoeal disease	South Asia	No	No	Yes	12.04	42.9	174	17.4	40	69	207	30
Control	Diarrhoeal disease	Africa	Yes	No	Yes	16.08	45.3	238	1.1	18	11	152	7.4
Control	Acute respiratory infection	South Asia	No	Yes	Yes	5.26	40.6	190	0.5	15	12	152	22.2
Control	Malaria	Africa	Yes	No	Yes	2.12	35.4	80	1.2	14	14	266	80.9
Control	Diarrhoeal disease	Southeast Asia	Yes	No	No	6.11	40.1	206	0.8	18	16	139	0.6
Control	Viral syndrome	Southeast Asia	No	No	No	4.81	36.2	230	0.7	234	217	1734	10.9
Control	Diarrhoeal disease	Africa	No	Yes	Yes	16.88	44.9	208	0.1	21	18	156	168.8
Control	Viral syndrome	South Asia	No	Yes	Yes	11.17	38.5	335	0.5	22	41	247	111
Control	Non-diarrhoeal gastrointestinal diagnosis	Oceania	No	No	No	10.52	39	284	0.6	30	72	178	161.9
Control	Dengue fever	South Asia	No	No	No	4.31	41	106	1.2	70	56	565	4
Control	Acute respiratory infection	Southeast Asia	No	No	Yes	4.66	42.5	172	0.6	17	9	159	28.6
Control	Diarrhoeal disease	Southeast Asia	Yes	Yes	Yes	8.56	38.2	207	0.6	25	14	175	8.9
Control	Acute respiratory infection	Oceania	No	No	Yes	3.7	34.6	147	0.4	19	12	149	65.9
Control	Viral syndrome	Africa	Yes	No	Yes	8.86	47.3	144	0.7	33	17	267	15.6
Control	Diarrhoeal disease	South Asia	Yes	No	Yes	14.39	41.6	214	3.1	23	16	199	2.9
Control	Acute respiratory infection	South America	Yes	No	No	6.83	39.5	209	0.8	17	14	156	9.1
Control	Acute respiratory infection	Southeast Asia	No	No	Yes	11.17	42.7	137	1.1	19	22	224	35.2
Control	Viral syndrome	Southeast Asia	Yes	No	Yes	6.12	45.5	162	0.7	22	19	246	14
Control	Acute respiratory infection	Southeast Asia	Yes	Yes	Yes	4.02	47.1	111	0.6	22	27	159	7.9
Control	Viral syndrome	Southeast Asia	No	Yes	Yes	14.67	45.1	152	0.5	92	149	471	11.2
Control	Diarrhoeal disease	Southeast Asia	Yes	No	No	11.04	42.2	275	0.8	12	18	151	10

Control	Acute respiratory infection	Southeast Asia	Yes	No	Yes	14.55	43	187	1.2	19	19	162	50.8
Control	Acute respiratory infection	South Asia	Yes	Yes	Yes	16.69	36.3	242	1	18	19	197	40.8
Control	Diarrhoeal disease	Southeast Asia	No	Yes	Yes	11.35	43.2	265	0.6	53	121	193	20.5
Control	Dengue fever	Southeast Asia	Yes	Yes	Yes	2.6	33.6	221	0.5	31	27	166	1.3
Control	Viral syndrome	Africa	No	No	Yes	8.68	51.7	231	1.1	19	17	168	37.1
Control	Diarrhoeal disease	South Asia	Yes	Yes	Yes	6.49	48.3	156	1	24	19	218	30.1
Control	Malaria	Africa	No	Yes	Yes	11.27	45.8	18	5	137	90	620	112.7
Control	Diarrhoeal disease	Southeast Asia	No	Yes	Yes	9.88	46.4	153	0.6	28	23	246	43
Control	Diarrhoeal disease	South Asia	No	No	No	6.79	43.3	187	0.5	27	26	278	5.6
Control	Diarrhoeal disease	Southeast Asia	Yes	No	Yes	8.03	45.6	292	0.5	44	111	229	0.6
Control	Viral syndrome	Southeast Asia	Yes	No	No	5.21	42.3	303	0.6	20	21	180	2.6
Control	Viral syndrome	South Asia	No	No	Yes	10	43.7	179	0.6	17	17	219	7.3
Control	Viral syndrome	Africa	Yes	No	No	7.87	43.5	218	1.1	24	27	200	50.2
Control	Diarrhoeal disease	South Asia	No	Yes	Yes	22.72	45.1	233	1.1	17	16	238	86.2
Control	Malaria	Africa	No	No	Yes	5.21	31.6	15	5.9	124	140	556	144
Control	Diarrhoeal disease	Southeast Asia	No	Yes	Yes	9.88	17.2	149	2.2	43	81	300	25
Control	Acute respiratory infection	Southeast Asia	Yes	No	Yes	9.5	44.4	259	0.5	23	27	307	40.6
Control	Acute respiratory infection	Southeast Asia	Yes	Yes	Yes	9.52	46.1	233	0.6	18	25	180	31.6
Control	Malaria	Africa	Yes	Yes	Yes	6.45	48.1	135	1.8	22	43	249	61
Control	Acute respiratory infection	South Asia	Yes	Yes	Yes	14.69	43.3	158	1.5	25	34	239	202
Control	Diarrhoeal disease	Southeast Asia	No	Yes	Yes	12.15	44.5	194	0.8	16	12	154	28.7

Control	Viral syndrome	Africa	Yes	No	Yes	6.43	42.3	148	1.7	18	15	168	14.6
Control	Leptospirosis	Oceania	No	Yes	Yes	8.26	43	182	1.3	20	23	203	194.4
Control	Malaria	Africa	Yes	No	No	3.48	46.2	108	0.7	19	31	261	46.6
Control	Leptospirosis	Southeast Asia	Yes	No	Yes	2.89	38.9	101	0.7	288	164	485	248.2
Control	Malaria	Africa	Yes	Yes	Yes	4.17	39	109	0.4	25	23	229	27.3
Control	Dengue fever	Southeast Asia	Yes	Yes	Yes	3.96	46.4	146	0.6	36	36	201	46.1
Control	Malaria	Africa	No	Yes	Yes	4.91	42.9	145	0.9	41	39	273	86
Control	Diarrhoeal disease	South Asia	Yes	No	Yes	6.42	45.1	182	1.1	19	17	166	25.3
Control	Malaria	Africa	No	Yes	Yes	5.56	54.8	101	2	50	76	334	38.9
Control	Acute respiratory infection	Southeast Asia	Yes	No	No	9.45	45.4	192	1.7	28	31	217	96
Control	Malaria	Africa	No	No	No	4.26	48.2	97	1.3	85	92	382	25.4
Control	Acute respiratory infection	Africa	No	No	Yes	3.6	46.1	161	0.6	27	29	218	1.3
Control	Acute respiratory infection	Southeast Asia	No	No	Yes	6.28	45.9	209	0.8	23	24	195	2.5
Control	Dengue fever	Oceania	Yes	Yes	Yes	2.6	43.9	74	0.4	85	28	513	1.1
Control	Acute respiratory infection	Africa	Yes	Yes	Yes	6.91	33.7	245	0.6	13	9	139	13.8
Control	Diarrhoeal disease	South Asia	No	No	Yes	6.33	41.5	193	0.9	19	21	178	21.2
Control	Acute respiratory infection	South Asia	Yes	No	Yes	15.93	46.9	277	0.6	38	35	279	131.9
Control	Rickettsiosis	Africa	No	No	No	5.66	50.1	141	0.6	40	47	324	83.9
Control	Viral syndrome	Africa	Yes	No	Yes	1.194	44.5	234	0.6	20	21	132	0.7
Control	Genitourinary infection	Southeast Asia	Yes	Yes	Yes	16.34	34.2	324	1.6	18	10	139	79
Control	Viral syndrome	Africa	No	No	No	5.98	40.1	264	0.4	19	10	220	2.5

Control	Viral syndrome	Southeast Asia	Yes	Yes	Yes	1.86	36.8	76	0.7	35	26	153	25.9
Control	Malaria	Africa	Yes	Yes	Yes	4.7	40.8	120	2.3	35	33	252	131.7
Control	Malaria	Africa	Yes	No	Yes	4.01	37.4	82	1.2	37	36	281	50.3
Control	Acute respiratory infection	South Asia	No	No	Yes	19.22	46.5	205	0.7	19	29	173	4
Control	Diarrhoeal disease	Southeast Asia	No	Yes	Yes	4.43	36.4	165	0.5	17	9	166	33.4
Control	Viral syndrome	Southeast Asia	No	Yes	Yes	6.13	33.7	150	1	22	21	175	6.9
Control	Dengue fever	Oceania	No	Yes	Yes	1.7	40.6	98	0.5	96	54	397	0.6
Control	Dengue fever	Southeast Asia	Yes	Yes	Yes	2.17	38.4	106	0.4	21	13	181	7.5
Control	Diarrhoeal disease	Southeast Asia	Yes	No	Yes	9.43	38.6	279	0.5	14	12	193	173
Control	Malaria	Africa	Yes	No	Yes	4.58	39.2	173	0.7	16	15	157	9.5
Control	Viral syndrome	Southeast Asia	No	No	Yes	1.342	42.2	185	2	19	12	181	14.9
Control	Diarrhoeal disease	Southeast Asia	Yes	No	Yes	6	40.8	255	2.1	27	27	206	7.9
Control	Dengue fever	Southeast Asia	Yes	No	No	2.5	41.5	38	0.4	51	25	362	7.1
Control	Non-diarrhoeal gastrointestinal diagnosis	Southeast Asia	Yes	No	No	5.94	42.9	136	6.1	102	339	295	84
Control	Viral syndrome	Southeast Asia	No	Yes	Yes	6.37	46	164	2.3	16	15	165	17.1
Control	Malaria	Africa	Yes	Yes	Yes	3.26	39	60	2.5	24	20	273	87.3
Control	Acute respiratory infection	Southeast Asia	No	No	Yes	15.49	45.4	196	1.4	40	67	236	147.7
Control	Non-diarrhoeal gastrointestinal diagnosis	Caribbean	No	No	No	16.61	43.9	260	0.5	29	52	203	51.3
Control	Acute respiratory infection	Africa	No	Yes	Yes	15.64	40.1	263	0.8	25	36	242	91.2
Control	Dengue fever	Southeast Asia	Yes	Yes	Yes	1.29	41.7	109	0.6	68	43	254	6.5
Control	Dengue fever	Southeast Asia	Yes	Yes	Yes	1.34	42.7	178	7	30	20	230	2.6

Control	Viral syndrome	Southeast Asia	Yes	No	No	6.42	36.9	157	0.7	27	32	227	51.9
Control	Non-diarrhoeal gastrointestinal diagnosis	Oceania	Yes	No	Yes	2.93	39.8	111	1	855	513	500	43.1
Control	Dengue fever	Southeast Asia	Yes	No	No	1.96	34.2	93	0.5	45	23	366	2.4
Control	Viral syndrome	Southeast Asia	Yes	Yes	Yes	3.8	43.5	162	0.3	108	106	604	56.2
Control	Diarrhoeal disease	Southeast Asia	No	No	Yes	12.17	40.8	209	0.7	40	75	167	15.4
Control	Genitourinary infection	Africa	No	No	Yes	8.63	34.5	250	0.8	48	405	202	89.6
Control	Diarrhoeal disease	Southeast Asia	Yes	No	No	8.15	43.8	204	0.8	25	52	178	8.2
Control	Acute respiratory infection	Africa	Yes	No	Yes	13.42	42.7	229	1.5	43	44	220	183.9
Control	Malaria	Africa	No	Yes	Yes	5.67	45.6	70	2	67	50	507	107.6
Control	Acute respiratory infection	South Asia	No	No	Yes	10.23	43.2	301	1	25	18	251	104.2
Control	Diarrhoeal disease	Southeast Asia	Yes	No	Yes	7.61	48.1	231	1.2	18	14	211	45.2
Control	Diarrhoeal disease	Central America	No	No	No	11.87	49.2	213	1	17	12	210	32
Control	Acute respiratory infection	Southeast Asia	Yes	Yes	Yes	1.421	45.7	328	1.1	18	14	235	40.1
Control	Acute respiratory infection	Africa	No	No	Yes	5.34	35.3	248	0.5	16	10	118	10
Control	Viral syndrome	Southeast Asia	No	No	No	7.65	40	379	0.8	25	22	189	0
Control	Malaria	Africa	Yes	No	Yes	4.58	39.2	173	0.7	16	15	157	9.5
