# Supplementary material for: Role of classic signs as diagnostic predictors for enteric fever among returned travellers: Relative bradycardia and eosinopenia
Source: PLoS One. 2017 Jun 23;12(6):e0179814. doi: 10.1371/journal.pone.0179814 (PMC5482448; doi:10.1371/journal.pone.0179814)
Supplement: S1 Table — (PDF) [file pone.0179814.s002.pdf]

S1 Table. Baseline characteristics of patients with typhoid fever and paratyphoid fever

Characteristics	Typhoid fever (n = 17)	Paratyphoid fever (n = 23)	p-value
Age (years), median (range)	28 (23–32)	36 (30–37)	
Male gender, n (%)	12 (71)	15 (65)	0.72
Ethnic origin, n (%)			
Asian	15 (88)	22 (96)	0.39
White	2 (12)	1 (4)	0.39
Travel destination, n (%)			
Southeast Asia	4 (24)	8 (35)	0.44
South Asia	13 (76)	15 (65)	0.44
Examination finding			
Relative bradycardia	15 (88)	20 (87)	0.65
Laboratory findings, median (IQR)			
Total leukocytes ($\times 10^3/\mu L$)	4.7 (3.4–5.7)	5.6 (4.4–6.9)	0.078
Absolute eosinopaenia (0/μL), n (%)	10 (59)	15 (65)	0.68
Haematocrit (%)	40 (36–41)	41 (38–43)	0.33
Platelets ($\times 10^3/\mu L$)	174 (120–190)	200 (165–247)	0.063
Total bilirubin (mg/dL)	0.5 (0.3–0.7)	0.5 (0.4–0.6)	0.89
AST (IU/L)	47 (33–71)	24 (19–40)	0.087
ALT (IU/L)	68 (29–131)	46 (29–81)	0.5
LDH (IU/L)	404 (360–520)	345 (257–425)	0.073
CRP (mg/L)	39 (25–102)	39 (31–64)	0.92

IQR: interquartile range; AST: aspartate transaminase; ALT: alanine transaminase; LDH: lactate dehydrogenase; CRP: C-reactive protein
