# Supplementary material for: Role of classic signs as diagnostic predictors for enteric fever among returned travellers: Relative bradycardia and eosinopenia
Source: PLoS One. 2017 Jun 23;12(6):e0179814. doi: 10.1371/journal.pone.0179814 (PMC5482448; doi:10.1371/journal.pone.0179814)
Supplement: S2 Table — (PDF) [file pone.0179814.s003.pdf]

S2 Table. Clinical diagnoses for study controls

Diagnosis	Patients, n
Diarrhoeal disease	
Acute, unspecified	27
Shigella sonnei	4
Campylobacter jejuni	1
Acute respiratory infection	
Viral, unspecified	18
Influenza virus	5
Bacterial pneumonia	2
Streptococcal pharyngitis	2
Viral syndrome	
Unspecified	17
Infectious mononucleosis	2
Measles	2
Aseptic meningitis	1
Malaria	
Plasmodium falciparum	14
P. ovale	1
P. malariae	1
Dengue fever	
Dengue without warning signs	9
Dengue with warning signs	2
Dengue shock syndrome	2
Non-diarrhoeal gastrointestinal diagnosis	
Hepatic abscess, amoebiasis	1
Acute hepatitis A	1
Cholangitis	1
Appendicitis	1
Genitourinary infection	
Pelvic inflammatory disease	1
Pyelonephritis	1

Dermatologic infection	
Cellulitis	1
