# Supplementary material for: Role of classic signs as diagnostic predictors for enteric fever among returned travellers: Relative bradycardia and eosinopenia
Source: PLoS One. 2017 Jun 23;12(6):e0179814. doi: 10.1371/journal.pone.0179814 (PMC5482448; doi:10.1371/journal.pone.0179814)
Supplement: S3 Table — (PDF) [file pone.0179814.s004.pdf]

S3 Table. Frequency of relative bradycardia and absolute eosinopenia in patients with enteric fever (cases) and controls

	Relative bradycardia	p-value[*]	Absolute eosinopenia (0/μL)	p-value[*]
Enteric fever, n (%)	35/40 (88)		25/40 (63)	
Diarrhoeal disease	15/32 (47)	<0.001[§]	12/32 (38)	0.035
Acute respiratory infection	12/27 (44)	<0.001[§]	8/27 (30)	0.008[§]
Viral syndrome	9/22 (41)	<0.001[§]	7/22 (32)	0.021
Malaria	10/16 (63)	0.043	8/16 (50)	0.39
Dengue fever	11/13 (85)	0.56	9/13 (69)	0.46

[*]p-values were analysed to compare enteric fever with each disease.

[§]p-values <0.01 using Bonferroni correction were regarded as statistically significant.
