# Supplementary material for: Role of classic signs as diagnostic predictors for enteric fever among returned travellers: Relative bradycardia and eosinopenia
Source: PLoS One. 2017 Jun 23;12(6):e0179814. doi: 10.1371/journal.pone.0179814 (PMC5482448; doi:10.1371/journal.pone.0179814)
Supplement: S4 Table — (PDF) [file pone.0179814.s005.pdf]

S4 Table. Logistic regression analysis of variables in the prediction of enteric fever diagnosis among enteric fever (cases) and each disease in controls

	OR (95% CI) for EF versus DD	OR (95% CI) for EF versus ARI	OR (95% CI) for EF versus VS	OR (95% CI) for EF versus Malaria	OR (95% CI) for EF versus DF
Relative bradycardia	7.93 (2.47–25.47)	8.75 (2.62–29.22)	10.11 (2.85–35.83)	4.20 (1.06–16.68)	1.27 (0.22–7.50)
Absolute eosinopenia (0/µL)	2.78 (1.06–7.26)	3.96 (1.39–11.26)	3.57 (1.19–10.75)	0.39 (0.52–5.37)	0.74 (0.19–2.83)

OR: odds ratio; CI: confidence interval; DD: diarrhoeal disease; ARI: acute respiratory infection; VS: Viral syndrome; DF: Dengue fever
